# The impact of the field of view (FOV) on image quality in MDCT angiography of the lower extremities

**DOI:** 10.1007/s00330-021-08391-x

**Published:** 2021-12-13

**Authors:** Nigar Salimova, Jan B. Hinrichs, Marcel Gutberlet, Bernhard C. Meyer, Frank K. Wacker, Christian von Falck

**Affiliations:** Department of Diagnostic and Interventional Radiology, Hannover Medical School, Carl-Neuberg-Strasse 1, 30625 Hannover, Germany

**Keywords:** Computed tomography angiography, Peripheral arterial disease, Lower extremity

## Abstract

**Objectives:**

To evaluate the impact of the reconstructed field-of-view (FOV) on image quality in computed-tomography angiography (CTA) of the lower extremities.

**Methods:**

A total of 100 CTA examinations of the lower extremities were acquired on a 2 × 192-slice multidetector CT (MDCT) scanner. Three different datasets were reconstructed covering both legs (standard FOV size) as well as each leg separately (reduced FOV size). The subjective image quality was evaluated for the different vessel segments (femoral, popliteal, crural, pedal) by three readers using a semi-quantitative Likert scale. Additionally, objective image quality was assessed using an automated image quality metric on a per-slice basis.

**Results:**

The subjective assessment of the image quality showed an almost perfect interrater agreement. The image quality of the small FOV datasets was rated significantly higher as compared to the large datasets for all patients and vessel segments (*p* < 0.05) with a tendency towards a higher effect in smaller vessels. The difference of the mean scores between the group with the large FOV and small FOV was 0.68 for the femoral level, 0.83 for the popliteal level, 1.12 for the crural level, and 1.08 for the pedal level. The objective image quality metric also demonstrated a significant improvement of image quality in the small FOV datasets.

**Conclusions:**

Side-separated reconstruction of each leg in CTA of the lower extremities using a small reconstruction FOV significantly improves image quality as compared to a standard reconstruction with a large FOV covering both legs.

**Key Points:**

• *In CT angiography of the lower legs, the side-separated reconstruction of each leg using a small field-of-views improves image quality as compared to a standard reconstruction covering both legs.*

• *The side-separated reconstruction can be readily implemented at every commercially available CT scanner.*

• *There is no need for additional hardware or software and no additional burden to the patient.*

## Introduction

Computed tomography angiography (CTA) of the lower extremity is a well-established imaging modality that allows a rapid and comprehensive diagnosis of the peripheral vessels in patients with arterial occlusive disease, aneurysms, sequelae of trauma, preoperative planning, and postoperative monitoring [1, 2]. The diagnostic performance of CTA is generally very robust at the iliac, femoral, and popliteal levels. However, maintaining diagnostic image quality for the assessment of below-the-knee arteries remains challenging, mainly due to a suboptimal vessel contrast and the small vessel size, especially in combination with vessel wall calcifications [3–5]. While there are numerous publications on the optimization of vessel contrast using an optimized bolus and a second scan of the calf and foot level, there is only very sparse literature concerning the challenges of small vessel sizes [4, 6]. As vessel diameters in the iliac level are up to five times larger than in the lower leg and foot, it becomes obvious that it is reasonable to optimize image reconstruction parameters for this demanding vessel segment.

In other parts of the body, it is well established that the adjustment of the reconstructed field-of-view (FOV) has a decisive impact on image quality as the full spatial resolution of the scanner cannot be utilized with a standard large “full-body” FOV. Alternatively, the reconstruction matrix size can be increased [7–10]. However, this is not possible in most standard clinical MDCT scanners.

Therefore, the aim of this study was to evaluate the impact of a smaller reconstruction FOV on the image quality in CTA of the lower extremities as compared to a standard reconstruction.

## Materials and methods

### Patient population

This study is a retrospective image and data analysis of CTA examinations of the lower limb performed at a tertiary care center from September 2019 to May 2020. In total, 110 CT examinations were screened for possible inclusion in the study. Exclusion criteria were the presence of osteosynthesis material and major amputation of the lower extremities. A total of 100 examinations in 97 patients (55 men, 42 women) with a mean age of 65 years (range: 23–91 years) were finally included in our study. The most common indication for performing CTA was chronic peripheral artery disease (PAD, *n* = 43). The clinical stage of the chronic limb ischemia according to the Fontaine classification was as follows: stage I: *n* = 0, stage IIa: *n* = 0, stage IIb: *n* = 10, stage III: *n* = 20, stage IV: *n* = 13. Other indications included acute lower limb ischemia (*n* = 26), trauma (*n* = 6), postoperative controls after vascular surgery (*n* = 10), and complications after percutaneous vascular interventions (*n* = 15). The local ethics committee and the data protection officer approved our study. Written informed consent was obtained from the patients for anonymized data analysis.

### Scan protocol

All CTA examinations were performed with a 2 × 96 detector row MDCT scanner acquiring 2 × 192 slices using a diagonal flying focal spot (SOMATOM Force, Siemens Healthineers). The examinations were acquired with a standardized scan protocol with the patient positioned in the supine position. The image protocol is given in Table 1. A tourniquet was placed on both thighs to avoid early contrasting of the venous vessels. The scan area included the abdominal aorta and the complete peripheral run-off. CTA was performed in two spirals: first spiral from the diaphragm to the sole of the foot and the second spiral from mid-thigh to the sole of the foot. Contrast media (100 ml iomeprol 400 mg/ml; Iomeron 400, Bracco) was intravenously injected using a dual-syringe injector (Stellant, Medrad) via a peripheral venous catheter at a flow rate of 4.5 ml/s followed by a bolus of 50-ml physiological saline solution. The first acquisition was started automatically 3 s after the threshold of 250 ΔHU was reached in the descending aorta. The second spiral from mid-thigh to the sole of the foot was started with an interscan delay of 5 s. The tube voltage and current were automatically adjusted to patient weight and height using the scanner dose-optimization features.

**Table 1:** Scan and reconstruction parameters for the CT angiography of the lower extremities

Scan parameters	
Collimation	2 × 192 × 0.6 mm (2 × 96 detector rows with a diagonal flying focal spot)
Rotation time	0.25 s
Pitch factor	0.35
Reconstructed slice thickness	0.6 mm
Reconstruction increment	0.4 mm
Contrast volume	100 ml (400 mg/ml)
Saline chaser	50 ml
Flow	4.5 ml/s

In all examinations, side-separated reconstructions with a small FOV were calculated with a matrix size of 512 × 512 mm in addition to the standard reconstructions with “large” standard FOV as part of the routine clinical protocol established in our institution. A standard kernel (Bv40) was used for both reconstructions.

### Image analysis

#### Subjective

For data analysis, the vascular system was divided into the following anatomical segments: femoral vessels (common femoral artery, superficial femoral artery, deep femoral artery), popliteal artery, crural vessels (tibioperoneal trunk, fibular artery, anterior tibial artery, posterior tibial artery to the upper ankle), pedal vessels (dorsal pedis artery, posterior tibial artery below the upper ankle, medial, and lateral plantar artery).

The subjective analysis of the datasets was independently evaluated by three radiologists (two experienced radiologists and one resident radiologist) using a designated diagnostic workstation (Visage 7.1, Visage Imaging) and a medical-grade diagnostic monitor (RadiForce, EIZO Europe GmbH) based on a 5-point Likert scale (1 = non-diagnostic, 2 = poor, 3 = moderate, 4 = good, 5 = excellent) [11]. Datasets were presented in random order. The radiologists were not blinded to the type of reconstruction as the FOV size could be recognized from the images. Segments were considered non-diagnostic when there was no vascular contrast in that specific vessel so that the delineation or depiction of the vessel could not be judged. The readers were instructed to exploit the full range of the Likert scale. For image assessment, the readers were able to use average intensity projections (AvIP), thin-slice multiplanar reformats (MPR) in axial, coronal, sagittal, or oblique orientation, and maximum intensity projections (MIP). The readers were able to vary the slab thickness of the MIP images at their own discretion. However, it was mandatory to review each dataset at least once using axial thin slices.

#### Objective

**Fig. 1:**
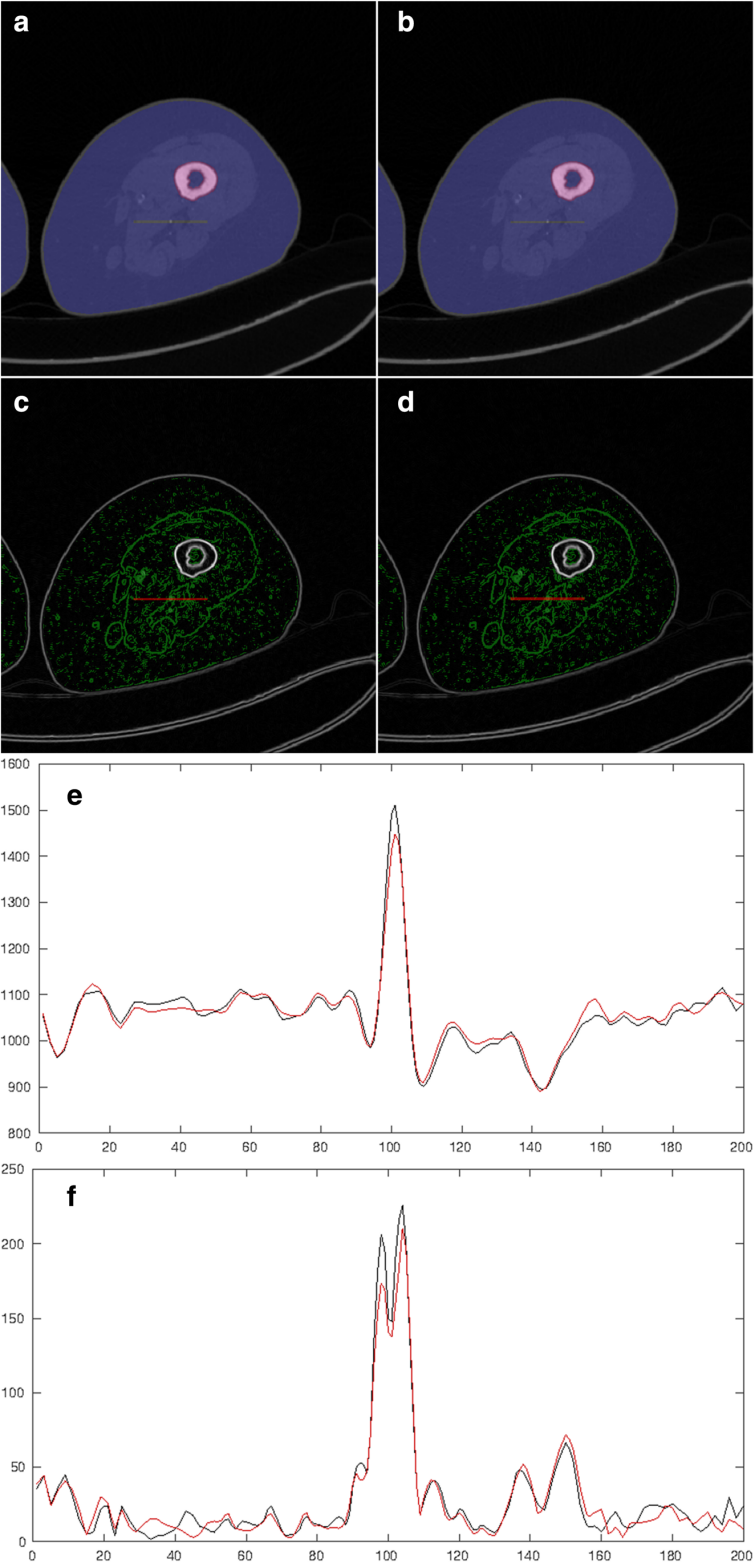
The objective image quality metric. First, the bone (red) and soft tissues (blue) are automatically segmented for the standard FOV (**a**) and the small FOV (**b**). Afterward, the significant edges are automatically identified (**c, d**) and the 2D image gradient is calculated on a slice-by-slice basis. An exemplary line profile (**e**) and image gradient profile (**f**) for the small (black line) and standard (red line) FOV is calculated across a major vessel (red line in **a–d**), demonstrating the improvement of image sharpness for the small FOV reconstruction

The objective evaluation and comparison of image sharpness of the imaged anatomical structures were automated using the square root of the sum of squares of the image gradient in both spatial dimensions summed over the region of interest on a slice-by-slice basis. To compare the image sharpness of small structures, sharp edges like at the object to air and tissue to bone boundaries were excluded. Therefore, an automated segmentation of the anatomy using Otsu’s thresholding method and including only the corresponding largest connected volume and of the bones was applied [12]. A subset of 17 datasets with almost perfect bone removal results was eligible for further analysis. To reduce the impairment of the image sharpness metric by noise, only significant edges were included using Otsu’s thresholding method applied to the magnitude of the spatial gradient of the images. The images of the large FOV were located at the exactly same voxel positions as of the small FOV using b-spline interpolation. The evaluation was performed with identical masks for the compared reconstructions. The automated objective image quality metric was implemented in Matlab R2020b [13]. An illustration of the approach is given in Fig. 1.

### Statistical analysis

The inter-rater agreement (inter-rater reliability) was analyzed using the intraclass correlation coefficient (ICC) with ICC values below 0.5 indicating poor reliability, values between 0.5 and 0.75 indicating moderate reliability, values between 0.75 and 0.9 indicating good reliability, and values above 0.90 indicating excellent reliability [14]. The ICC was calculated based on a “two-way random” model with mean score and absolute agreement (ICC2k) which was most appropriate for the given data [15].

A comparison of ordinal scaled variables between groups with large and small FOV was performed using the Wilcoxon signed-rank test. Differences between the right and left sides were assessed using the Wilcoxon rank-sum test. We also calculated mean values and standard deviations for the Likert scale data and tested using Student’s *t*-test as proposed in the literature [16]. Differences in FOV size as well as the data from the objective image quality metric were analyzed using Student’s *t*-test.

A *p* value < 0.05 was considered statistically significant for all tests. The statistical analysis and graph generation were performed using R 3.6.3 and RStudio 1.2.5042 [17].

## Results

The size of the FOV of the standard reconstruction (diameter: 422.67 mm ± 36.88 mm) was significantly higher (*p* < 0.05) as compared to the FOV of the side-specific reconstruction (diameter: 220.19 mm ± 30.63 mm), resulting in a mean in-plane voxel size of 0.83 mm and 0.43 mm, respectively.

A total of 1600 vessel segments (right and left) were evaluated. There was a higher number of segments rated non-diagnostic in the large FOV group (reader 1: 6, reader 2: 7, reader 3: 7) as compared to the small FOV group (reader 1: 4, reader 2: 0, reader 3: 4), respectively. All non-diagnostic segments were located at the crural and pedal level and excluded from further analysis.

The inter-rater agreement of all subjective measurements (scores) and all readers was considered ‘almost perfect’ according to Landis and Koch with an ICC value of 0.82 [14]. Therefore, the results of the three readers were pooled for further analysis.

**Fig. 2:**
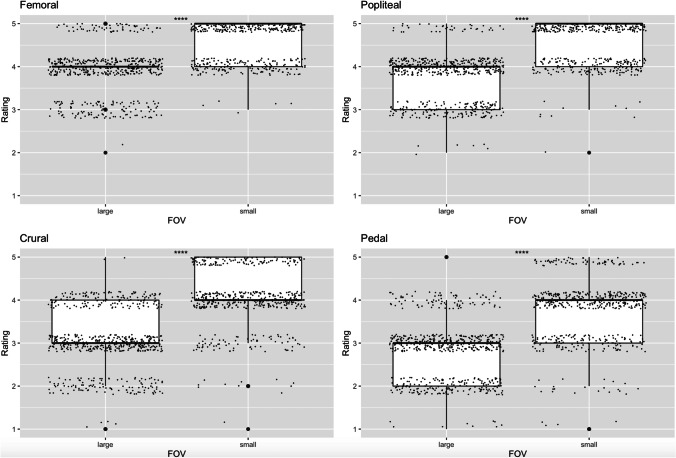
Box-plots of the image quality ratings for the small FOV versus the large FOV datasets at the different vessel segments. The ratings are averaged over the three readers. Random jitter was added to the datapoints to illustrate the frequency of the different reader scores. The improvement of the image quality using the small FOV reconstruction is clearly demonstrated. All results were statistically significant (*p* < 0.05)

There was a statistically significant difference in image quality between the small and large FOV groups for all vessel segments studied (femoral vessels, A. poplitea, crural vessels, pedal vessels) in both side-separated and cumulative data analysis (*p* < 0.05). The mean value of image quality using the 5-level Likert scale for the femoral vessels was 4.02 ± 0.65 (median = 4 [4]) for the large FOV and 4.70 ± 0.48 (median = 5 [4, 5]) for the small FOV for both locations (right and left); for the popliteal artery, the values were 3.82 ± 0.63 (median = 4 [3, 4]) for the large FOV and 4.65 ± 0.52 (median = 5 [4, 5]) for the small FOV; for the crural vessels 3.06 ± 0.72 (median = 3 [3]) for the large FOV and 4.18 ± 0.72 (median = 4 [4]) for the small FOV, for the pedal vessels 2.79 ± 0.73 (median = 3 [2, 3]) for the large FOV, and 3.87 ± 0.82 (median = 4 [3, 4]) for the small FOV. The difference of the mean values between the group with the large FOV and the small FOV was 0.68 for the femoral vessels, 0.83 for the popliteal artery, 1.12 for the crural vessels, and 1.08 for the pedal vessels. The results are summarized in Table 2 and illustrated in Fig. 2.

**Table 2:** Mean scores (± standard deviation) and median scores (with interquartile range) of the subjective image analysis. For each vessel segment the differences between the large and the small FOV were statistically significant (^*^Student’s *t-*test, ^#^Wilcoxon signed-rank test, *p* < 0.05)

Vessel segment	FOV size				
	Large		Small		Δ Mean
	Mean ± SD	Median	Mean ± SD	Median	
Femoral	4.02^*^ ± 0.65	4 [4-4]^#^	4.70^*^ ± 0.48	5 [4, 5]^#^	0.68
Popliteal	3.82^*^ ± 0.63	4 [3, 4]^#^	4.65^*^ ± 0.52	5 [4, 5]^#^	0.83
Crural	3.06^*^ ± 0.72	3 [3-3]^#^	4.18^*^ ± 0.72	4 [4-4]^#^	1.12
Pedal	2.79^*^ ± 0.73	3 [2, 3]^#^	3.87^*^ ± 0.82	4 [3, 4]^#^	1.08

In order to avoid a potential bias of the trauma patients with a possibly better image quality of these otherwise healthy patients, we separately analyzed this cohort and found the scores well within the range of the other patients (femoral large FOV: 3.83, femoral small FOV: 4.67; popliteal large FOV: 3.50, popliteal small FOV: 4.67; crural large FOV: 2.67, crural small FOV: 3.83; pedal large FOV: 2.69, pedal small FOV: 3.68).

In the analysis of image quality between the right and left leg, there was no statistically significant difference for all vessel segments regardless of FOV size (femoral large FOV (*p* = 0.78), femoral small FOV (*p* = 0.95); popliteal large FOV (*p* = 0.69), popliteal small FOV (*p* = 0.67); crural large FOV (*p* = 0.96), crural small FOV (*p* = 0.14); and pedal large FOV (*p* = 0.42), pedal small FOV (*p* = 0.49).

**Fig. 3:**
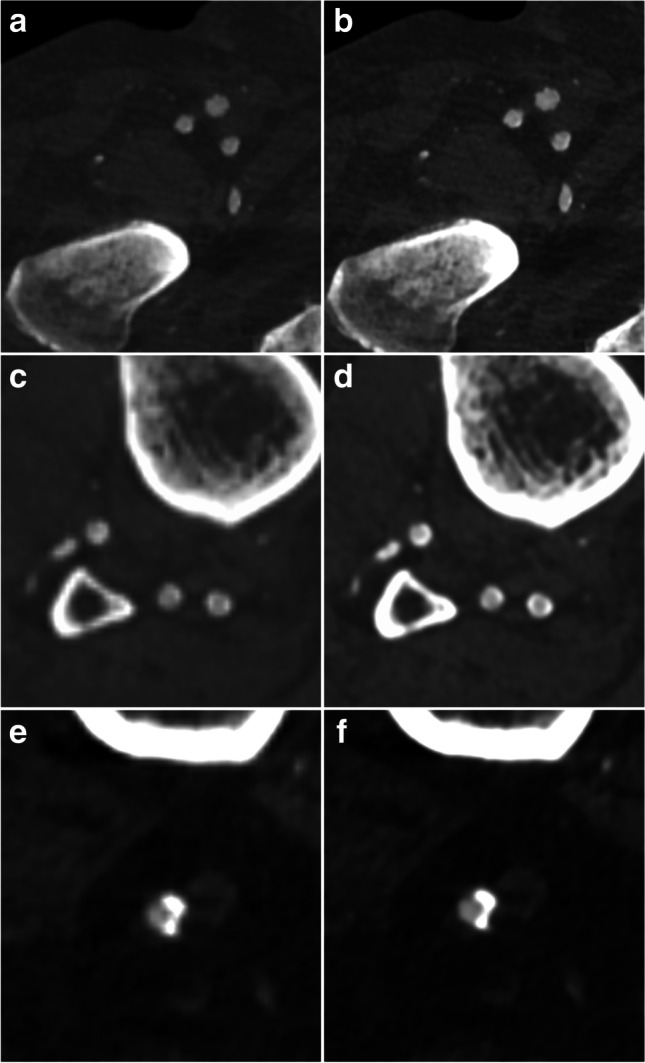
Illustration of the improvement of image quality for the small FOV reconstruction (right column) as compared to the standard reconstruction (left column). Already at the femoral level (**a**, **b)** a sharper image quality and better delineation of the vessel wall is noted. The effect is much more pronounced at the calf level (**c**, **d**) in the same patient with heavily calcified arteries. In another patient with severe vessel calcifications of the popliteal artery (**e**, **f**), the reconstruction with a smaller FOV results in a better delineation of the contrasted vessel lumen against the coarse calcification

Typical clinical examples that demonstrate the improvement of image quality at different vessel segments are shown in Fig. 3.

Using the objective metric, we found a superior sharpness of the anatomical structures for the small FOV as compared with the large FOV in all analyzed datasets. The mean difference of the image quality metric for the small FOV as compared with the large FOV over all slices and datasets was 2.1 (range 1.19–3.08). The results were statistically significant for all datasets (*p* < 0.05, unpaired one-sided *t*-test).

## Discussion

In this study, we were able to demonstrate that the use of a dedicated reconstruction of each leg using a small FOV in CTA run-off examinations of the lower extremities significantly increases the diagnostic image quality regardless of vessel size. Furthermore, it could be shown, that the effect is more pronounced at the below-the-knee level. This effect is due to a reduction of the in-plane voxel dimensions, resulting in higher spatial resolution and therefore better delineation of the vessel wall, especially in the presence of vascular calcifications. The low number of non-diagnostic vessel segments for both reconstructions indicates a high overall image quality.

We observed a benefit of a smaller FOV using both, a subjective and an objective image quality measure. The subjective analysis clearly showed a significant improvement of image quality for the small FOV at every vessel level at an “almost perfect” intraclass correlation. The objective image quality analysis confirmed the results by demonstrating a superior sharpness of the anatomical structures of interest (i.e., vessels) for the small FOV reconstruction.

The improvement of spatial resolution in MDCT using a smaller, adjusted FOV, or an increased matrix is well established in other parts of the body [7–10, 18]. Whereas most of these applications focus on high contrast structures such as the scaphoid or temporal bone, small joints, or the lungs, there is a prominent example for the use of a small FOV for vessels examinations in MDCT: cardiac CT [19, 20]. It is obvious, that (besides a high contrast-to-noise ratio) the spatial resolution is of decisive importance at the (distal) below-the-knee level as the size of the vessels to be analyzed approximates the in-plane voxel dimensions when using a standard FOV (approximately 0.8 mm). In accordance with this assumption, we found a more pronounced effect of the side-specific reconstruction on image quality for the crural and pedal levels.

A reduction of voxel dimensions is also achievable by increasing the reconstruction matrix (e.g., 1024 × 1024) [7, 10]. However, this option is not readily available in most clinical MDCT scanners and may hinder the use of standard PACS infrastructure (e.g., software compatibility). In contrast, the side-specific reconstruction of the below-the-knee level using a smaller FOV can be easily implemented at every CT scanner and does not require any additional hard- or software. Apparently, however, the number of images to be read and archived may increase, depending on the actual image reconstruction concept.

Improving the diagnostic performance of CTA of the lower extremities is of high clinical relevance as CTA has become a widely used standard procedure for the evaluation of patients with diseases of the peripheral vessels [1, 4, 21]. It has a proven high diagnostic sensitivity and specificity (> 95%) when compared with the invasive gold standard of digital subtraction angiography (DSA) [2, 3, 22]. Furthermore, CTA is increasingly used to guide treatment decisions (surgical vs. transluminal) and interventional treatment planning [23, 24]. Due to its superior clinical performance and robustness, CTA of the lower extremities is increasingly used in critical patients with PAD such as patients with impaired renal function and diabetes. In these examinations, image quality may be deteriorated as the amount of intravenously administered contrast agent is limited to reduce the risk of contrast-induced nephropathy. Furthermore, there is a higher prevalence of vessel wall sclerosis in this cohort that hinders the evaluation of the patency of small vessels [5, 25]. In these patients, the use of a side-specific reconstruction may increase image quality and add to a precise non-invasive diagnostic in this vulnerable patient group.

Several approaches have been proposed to improve image quality in CTA of the lower extremities, such as contrast bolus and timing optimization [26, 27], dynamic below-the-knee scanning [6], low-kV imaging, dual-energy techniques, and iterative or AI-based reconstructions [1]. Notably, the optimization of the reconstruction FOV as outlined in this publication can be readily combined with any of these approaches to further improve the performance of CTA run-off examinations.

There are several limitations of this study that need to be addressed. First of all, due to its retrospective nature, our study was limited to the evaluation of image quality rather than evaluating the impact of the improved image quality on clinical decision management. However, as non-invasive imaging such as peripheral run-off CTA is already the diagnostic modality of choice in patients with diseases of the peripheral, arteries there was no invasive gold standard (DSA) available in our patient cohort. Therefore, we were not able to calculate the improvement of diagnostic accuracy using the side-specific reconstruction as compared to the standard reconstruction. However, as the beneficial effect of the reconstruction of a smaller FOV was pronounced at the challenging below-the-knee level, it seems reasonable to assume that the improvement of image quality may translate into a clinical benefit.

Secondly, the objective analysis of image quality had to be limited to 17 datasets as the algorithm demands for a highly accurate segmentation of bony structures which only could be achieved in the minority of datasets. Further optimization of our automated approach might allow for a more robust segmentation and analysis of more CT data. Thirdly, although an optimized study protocol can be easily implemented at virtually every modern CT scanner, we are well aware that this may result in additional data burden that has to be handled. Furthermore, we did not analyze different reconstruction kernels or other scan parameters that might offer further potential for image quality improvement of run-off CTA. However, it seems reasonable to assume that side-specific reconstruction will improve quality independent of the reconstruction algorithm as the underlying physical effect is identical.

In conclusion, we were able to demonstrate that the simple optimization of the reconstruction FOV significantly improves the image quality in run-off CTA of the lower extremities, especially at the below-the-knee level.

## Abbreviations

AVIP: Average intensity projection
CT: Computed tomography
CTA: CT angiography
DSA: Digital subtraction angiography
FOV: Field-of-view
HU: Hounsfield unit
ICC: Intraclass correlation
kV: Kilovolt
mAs: Milliampere-seconds
MDCT: Multidetector computed-tomography
mg: Milligrams
MIP: Maximum intensity projection
ml: Millilitre
MPR: Multiplanar reformation
PACS: Picture Archiving and Communication System
PAD: Peripheral artery disease
s: Seconds

## Declarations

### Guarantor

The scientific guarantor of this publication is Christian von Falck.

### Statistics and biometry

No complex statistical methods were necessary for this paper.

### Methodology

• retrospective

• diagnostic study

• performed at one institution
